# Fine-Tuning Pyridinic Nitrogen in Nitrogen-Doped Porous Carbon Nanostructures for Boosted Peroxidase-Like Activity and Sensitive Biosensing

**DOI:** 10.34133/2020/8202584

**Published:** 2020-11-06

**Authors:** Hongye Yan, Linzhe Wang, Yifeng Chen, Lei Jiao, Yu Wu, Weiqing Xu, Wenling Gu, Weiyu Song, Dan Du, Chengzhou Zhu

**Affiliations:** ^1^Key Laboratory of Pesticide and Chemical Biology of Ministry of Education, International Joint Research Center for Intelligent Biosensing Technology and Health, College of Chemistry, Central China Normal University, Wuhan 430079, China; ^2^State Key Laboratory of Heavy Oil Processing, China University of Petroleum, Beijing 102249, China; ^3^School of Mechanical and Materials Engineering, Washington State University, Pullman, Washington 99164, USA

## Abstract

Carbon materials have been widely used as nanozymes in bioapplications, attributing to their intrinsic enzyme-like activities. Nitrogen (N)-doping has been explored as a promising way to improve the activity of carbon material-based nanozymes (CMNs). However, hindered by the intricate N dopants, the real active site of N-doped CMNs (N-CMNs) has been rarely investigated, which subsequently retards the further progress of high-performance N-CMNs. Here, a series of porous N-CMNs with well-controlled N dopants were synthesized, of which the intrinsic peroxidase (POD)like activity has a positive correlation with the pyridinic N content. Density functional theory calculations also reveal that pyridinic N boosts the intrinsic POD-like activity of N-CMNs. Pyridinic-N dopant can effectively promote the first H_2_O desorption process in comparison with the graphitic and pyrrolic N, which is the key endothermic reaction during the catalytic process. Then, utilizing the optimized nanozymes with high pyridinic N content (N_P_-CMNs) and superior POD-like activity, a facile total antioxidant capacity (TAC) assay was developed, holding great promise in the quality assessment of medicine tablets and antioxidant food for healthcare and healthy diet.

## 1. Introduction

Nanomaterial-based enzyme mimics, defined as nanozymes, have emerged and attracted considerable attention recently. As emerging substitutes of natural enzymes, nanozymes possess exceptional features including high stability, durability under harsh conditions, and simple preparation with low costs [1–9]. Carbon material-based nanozymes (CMNs), as a star family in nanozymes, have been widely explored in sensing, therapy, and catalysis, since they have been discovered to possess high operational stability against stringent conditions and could be prepared facilely with low costs [10–16]. However, as metal-free catalysts, pure CMNs have been endowed with moderate catalytic activities, which are still far from meeting the requirements of high catalytic activity. To address this issue, heteroatom (N, B, P, S, or Se) doping is an efficient method to improve the catalytic activities of carbon materials [17–23]. Among them, N-doping has been extensively studied due to the fact that a similar atomic radius of nitrogen as carbon makes it easier to incorporate into the graphitic lattice [10, 24].

To date, N-doped CMNs (N-CMNs) have shown great promise in biosensors, clinical diagnosis, and therapy, attributing to their intrinsic peroxidase (POD)-like activities [10–13]. Although great progress has been made in this field, further verification of active sites and in-depth understanding of their catalytic mechanisms are still required. It is well established that nitrogen dopants play a key role in boosting the activities of carbon materials in different catalytic reactions [25–30]. However, few efforts have been devoted to figuring out the relationship between different N species (pyridinic, pyrrolic, graphitic, and oxidized N) and enzyme-like activities of N-CMNs, which retards further development of high-performance N-CMNs and deeper understanding of structure-activity relationships of the catalyst at the atomic level. Due to the close formation energy of the N species, model nanozymes with well-controlled N dopants are still extremely challenging to obtain [31]. Towards this end, there is an urgent need to design model N-CMNs with modulated N dopants and identify the active sites of N-CMNs, enabling an in-depth understanding of the nature of the catalysis and bringing unprecedented insights into the rational design of N-CMNs.

Herein, taking N-CMNs with well-controlled N dopants as model nanozymes, we present a successful paradigm to throw new light on the structure-activity relationship of N-CMNs. By secondary N doping, N_P_-CMNs were synthesized with porous structures and high pyridinic N content, which possessed superior POD-like activity. Unexpectedly, there was a positive correlation between the pyridinic N content and the intrinsic POD-like activity of N-CMNs. Theoretical calculations reveal that the pyridinic N dopant plays a key role in boosting the intrinsic POD-like activity of N-CMNs. Compared with graphitic and pyrrolic N, pyridinic N can significantly reduce the endothermic reaction energy of the first H_2_O desorption step which is the key elementary step of the catalytic process. On this basis, N_P_-CMNs were used to develop a sensing platform devoting to the assessment of the total antioxidant capacity (TAC), which is a key factor to evaluate the oxidative stress in healthcare and the food quality [32].

## 2. Results and Discussion

**Figure 1:**
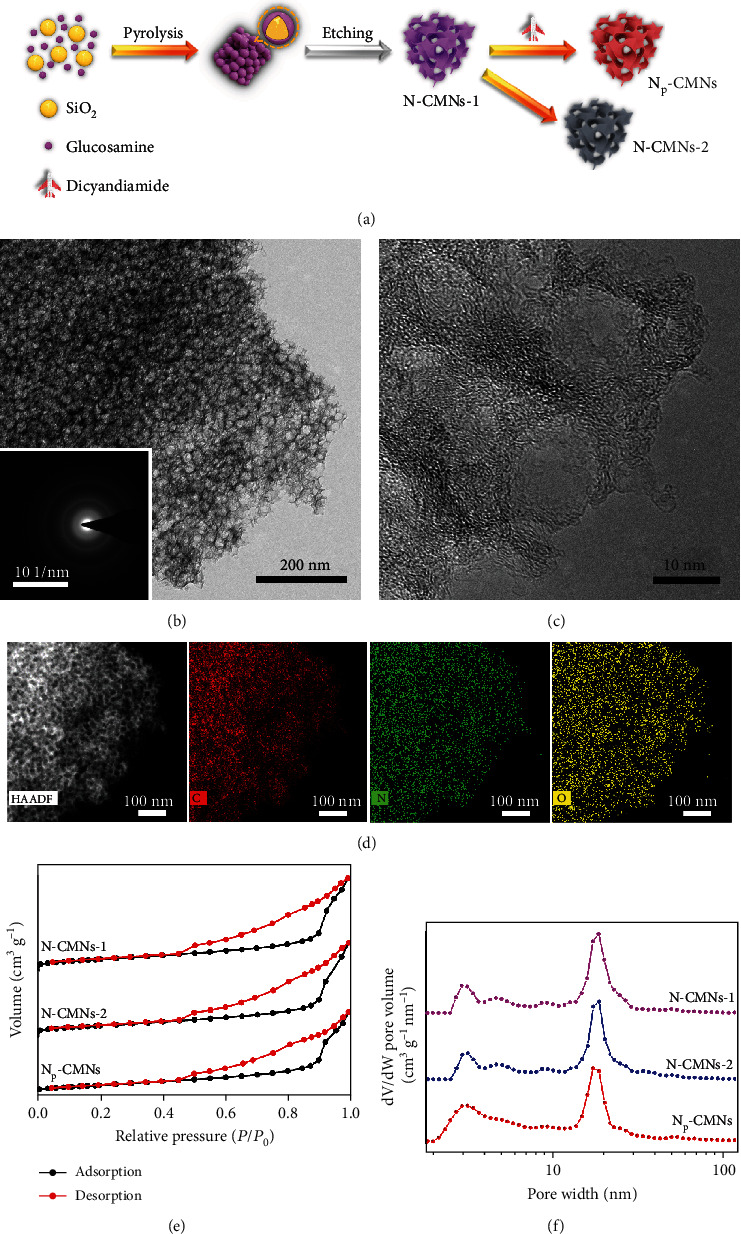
(a) Schematic illustration of the synthesis of N-CMNs. TEM image (b), SAED pattern (b, inset), HRTEM image (c), and HAADF-STEM image and the corresponding EDS mappings (d) of N_P_-CMNs. N_2_ physisorption isotherm (e) and pore width distribution (f) of different N-CMNs.

Using silica colloid as hard templates, N-CMNs-1 were synthesized via a pyrolysis process in the presence of glucosamine, targeting the simultaneous formation of porous structures and dispersed N dopants (Figure 1(a)). After secondary annealing treatment, N_P_-CMNs were obtained using dicyandiamide as secondary nitrogen sources. As a control, N-CMNs-2 were synthesized in the same manner without nitrogen sources. Given that dicyandiamide is decomposed to ammonia (NH_3_) gas during annealing, the released NH_3_ can benefit the transformation of N species, achieving the modulation of nitrogen doping [33, 34]. The transmission electron microscopy (TEM) image of the as-prepared N_P_-CMNs is shown in Figure 1(b), which clearly displays the chiseled spherical pores with a diameter of 20 nm, deriving from the silica templates. The ring-like selected area electron diffraction (SAED) pattern (Figure 1(b), inset) illustrates the amorphous nature of N_P_-CMNs. Besides, as can be seen from the high-resolution TEM (HRTEM) image in Figure 1(c), N_P_-CMNs consist of the curved graphene layers with a low crystallinity deduced from the unclear lattice fringes of graphitic carbon, which is consistent with the SAED result. As displayed in Figure S1, the introduction of secondary nitrogen sources makes no difference to the morphology of N-CMNs. High-angle annular dark-field scanning TEM (HADDF-STEM) image and the corresponding energy disperse spectroscopy (EDS) mappings (Figure 1(d)) demonstrate the homogeneous distribution of C, N, and O elements in the nanozymes. As shown in Figure 1(e), all the nanozymes exhibit obvious hysteresis loops in N_2_ adsorption-desorption isotherms, indicating their mesoporous properties ascribed to the silica template packing. The Brunauer-Emmett-Teller (BET) surface areas are 322.3, 309.2, and 302.5 m^2^ g^−1^ for N-CMNs-1, N-CMNs-2, and N_P_-CMNs, respectively, indicating that the second pyrolysis has no obvious effect on their morphologies and surface areas.  Figure 1(f) shows that the pore width of the nanozymes ranges from 2 to 30 nm (mesopores), in which most pores range around 20 nm with a normal distribution resulting from the silica templates. The hierarchical mesopores of N-CMNs not only increases the density of exposed active sites but also renders a faster mass transport, which is beneficial for further enhancing the nanozyme activities.

**Figure 2:**
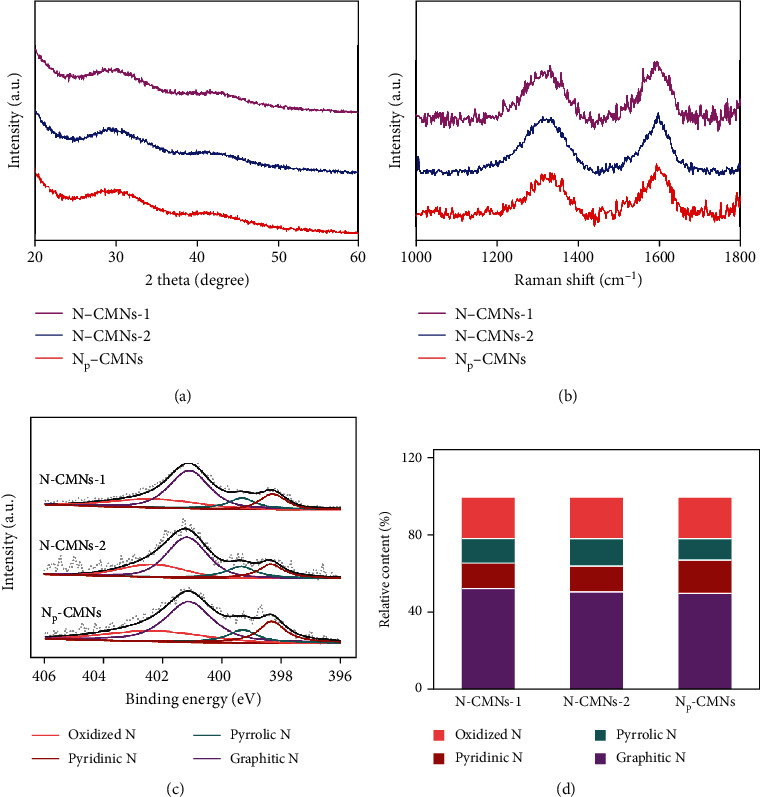
XRD patterns (a) and Raman spectra (b) of N-CMNs-1, N-CMNs-2, and N_P_-CMNs. N 1s high-resolution XPS spectra (c) and the corresponding relative contents of different N species (d) of the as-prepared nanozymes.

X-ray powder diffraction (XRD) was investigated to assess the graphitization degree of the as-prepared N-CMNs. As shown in Figure 2(a), two broad diffraction peaks of N-CMNs centered at 2*θ* ≈ 30 and 42 ° can be indexed to carbon (002) and (101), respectively, demonstrating the poor crystalline structure of the nanozymes [9, 35]. Moreover, the nanozymes display the main Raman features of the D band at 1310 cm^−1^ and G band at 1590 cm^−1^ (Figure 2(b)). The peak intensity ratios of the D and G bands (I_D_/I_G_) of the nanozymes are 0.92 (N-CMNs-1), 0.94 (N-CMNs-2), and 0.90 (N_P_-CMNs), respectively, suggesting all of them possess the similar defect density resulting from the doping of N atoms into the graphene network [36]. Together, in spite of the different pyrolysis procedures, no significant changes in the morphology and the crystalline structures of N-CMNs were observed from the various characterizations above. Then, X-ray photoelectron spectroscopy (XPS) analyses were performed to identify the elemental compositions of N-CMNs, confirming the existence of C, N, and O (Figure S2). N-CMNs synthesized with different N doping levels offer ideal models to investigate the role of N dopants in the catalytic process. As shown in Table S1, all N-CMNs possess high atom ratios of total nitrogen (5.85-6.21%), which is higher than those of some reported N-doped carbons [10, 30, 35]. The configuration of the doped N species was further determined by deconvolution N 1s spectra (Figure 2(c)). There are four typical peaks at 398.33, 399.35, 401.15, and 402.33 eV, assigned to pyridinic, pyrrolic, graphitic, and oxidized N, respectively. It is found that graphitic N is the predominant peak in N 1s XPS spectra, while pyrrolic, graphitic, and oxidized N for the three types of N-CMNs were observed with similar content, respectively (Figure 2(d) and Table S2). Note that the atomic percent of pyridinic N of N_P_-CMNs is 1.02%, remarkably higher than those of N-CMNs-1 (0.828%) and N-CMNs-2 (0.832%), which is expected to exert great influence in the nanozyme activities.

**Figure 3:**
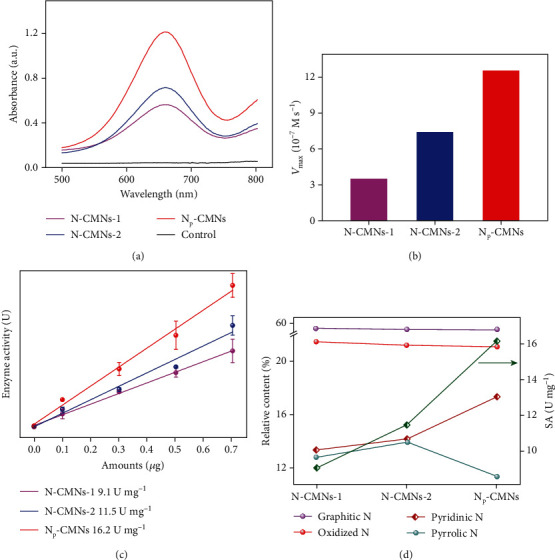
(a) UV-vis absorption spectra of POD-like catalytic oxidation in the presence of N-CMNs. Comparison of the *V*_max_ (b) (taking H_2_O_2_ as substrate) and the SA (c) of N-CMNs. (d) The correlation between the relative content of N dopants and the SA of N-CMNs.

The POD-like activity of N-CMNs was investigated and contrasted by monitoring 3, 3′, 5, 5′-tetramethylbenzidine (TMB) oxidation taking hydrogen peroxide (H_2_O_2_) as substrate. As shown in Figure 3(a), the solution containing N_P_-CMNs shows a much higher absorbance of oxidized TMB (oxTMB) at 652 nm than those of the solution containing N-CMNs-1 and N-CMNs-2, indicating that the POD-like activity of N_P_-CMNs is remarkably enhanced after secondary N-doping. As expected, the catalytic rates of the nanozyme-catalyzed reaction increase gradually and reach a plateau with the increase of substrate concentration (Figure S3), which suggests that the N-CMNs-catalyzed reactions obey the Michaelis-Menten kinetics. As shown in Figure 3(b) and Table S3, N_P_-CMNs exhibit the highest POD-like activity according to the values of the Michaelis-Menten constant (*K*_*M*_) and maximal velocity (*V*_max_), which are the key kinetic parameters for estimating the performance of nanozymes [37]. Meanwhile, the proposed N_P_-CMNs have competitive POD-like activities compared with other reported nanozymes (Table S4) [38–42]. In addition, the specific activity (SA), defined as activity unit (U) per milligram (mg) of nanozyme, was quantitatively contrasted to further investigate the POD-like activities of the N-CMNs (Figure 3(c)). The SA of N_P_-CMNs is 16.2 U mg^−1^, much higher than those of N-CMNs-1 (9.1 U mg^−1^) and N-CMNs-2 (11.5 U mg^−1^), indicating the superior activity of N_P_-CMNs. As can be seen from Figure 3(d), there is an unexpected positive correlation between the relative content of pyridinic N and the SA of N-CMNs, which suggests that the pyridinic N dopant plays a crucial role in improving the POD-like activities of N-CMNs. The optimized condition for the POD-like catalysis of N_P_-CMNs, including pH and temperature, were investigated (Figure S4). The activity of N_P_-CMNs reaches its maximum at pH 3.0, and N_P_-CMNs possess a high tolerance to the change of temperature in comparison with natural horseradish peroxidases (HRP).

**Figure 4:**
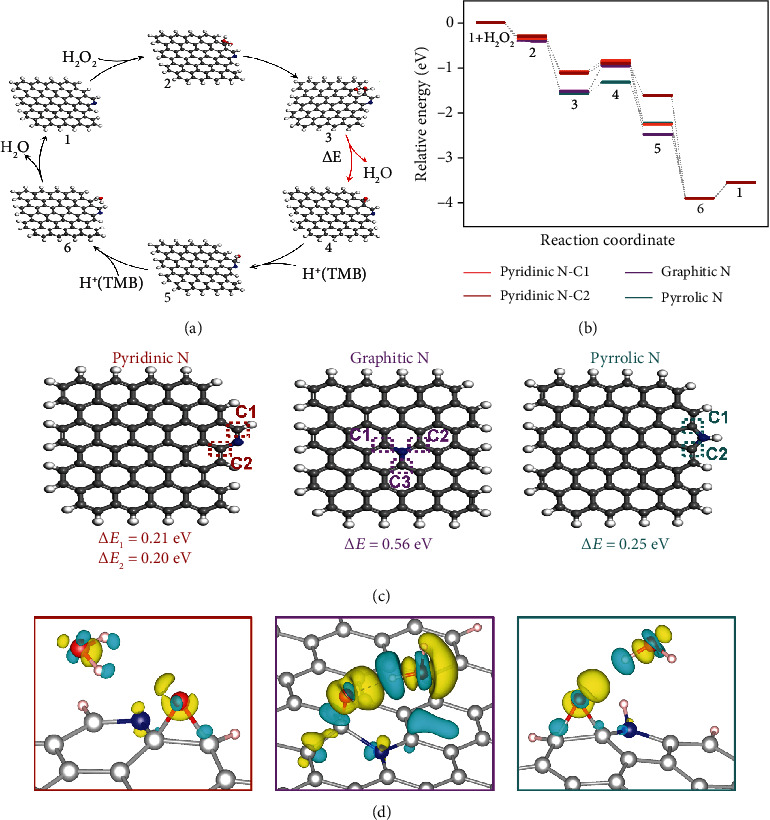
(a) The process of POD-like catalytic reaction on pyridinic N-doped model graphene. (b) Energy profile for POD-like reactions on three types of N-doped model graphene sheets. (c) The optimized structures of graphitic N, pyridinic N, and pyrrolic N models and the corresponding reaction energies of the key endothermic step 3. (d) Difference charge densities of H_2_O adsorption on the oxidation-state intermediate 3 (isosurface value is 0.008 e Å^−3^). The blue and yellow regions indicate the depletion and the accumulation of electronic charge, respectively.

To further elucidate the active sites and catalytic mechanism, POD-like activities on the three types of N-doped model graphene sheets (graphitic N, pyridinic N, and pyrrolic N models) were investigated by density functional theory (DFT) calculations. Notably, it is generally accepted that hydroxyl radicals (∙OH) are the active intermediates of the catalytic reactions involving carbon nanozymes [11]. Unexpectedly, as displayed in the electron spin resonance (ESR) spectra (Figure S5a), there is no ∙OH generated and captured during the catalytic process of N-CMNs. In addition, the p-phthalic acid (PTA) is used as a fluorescence probe of ∙OH while no characteristic emission of the fluorescent product near 430 nm is observed in Figure S5b, agreed well with the abovementioned result from the ESR spectra. In consequence, we speculate that the other high oxidation-state intermediates dominate the POD-like catalysis of N-CMNs, which is similar to the natural HRP [43]. Then, the catalytic process of N-CMNs is considered to proceed as the following six elementary steps [12]:

(1) $$\begin{array}{c} {\text{H}}_{2}{\text{O}}_{2}+*\to {\text{H}}_{2}{{\text{O}}_{2}}^{*} \end{array}$$

(2) $$\begin{array}{c} {{\text{H}}_{2}{\text{O}}_{2}}^{*}+*\to {\text{H}}_{2}{\text{O}}^{*}+{\text{O}}^{*} \end{array}$$

(3) $$\begin{array}{c} {\text{H}}_{2}{\text{O}}^{*}+{\text{O}}^{*}\to {\text{O}}^{*}+{\text{H}}_{2}\text{O}+* \end{array}$$

(4) $$\begin{array}{c} {\text{O}}^{*}+{\text{H}}^{+}+{\text{e}}^{-}\to {\text{HO}}^{*} \end{array}$$

(5) $$\begin{array}{c} {\text{HO}}^{*}+{\text{H}}^{+}+{\text{e}}^{-}\to {\text{H}}_{2}{\text{O}}^{*} \end{array}$$

(6) $$\begin{array}{c} {\text{H}}_{2}{\text{O}}^{*}\to {\text{H}}_{2}\text{O}+* \end{array}$$

where the asterisk indicates the adsorption site. Steps 1-3 are considered as the H_2_O_2_ decomposition steps, while steps 4 and 5 are the proton-electron pair transfer steps. As shown in Figure 4(a), taking the carbon atoms connected to N as the active sites, H_2_O_2_ is first adsorbed on N-doped model graphene, and then, a deoxidation process of H_2_O_2_ occurs, followed by the desorption of a H_2_O molecule and the formation of oxidation-state intermediate 4 in which the O atom is connected to the active site on the top site of graphitic N model and bridge site of pyridinic N and pyrrolic N models, respectively. On the premise of providing protons by substrate TMB, the two proton-electron pair transfer steps are sequentially achieved, followed by the formation and removal of H_2_O. Based on the above reaction path, the potential energy diagram was obtained in Figure 4(b), where steps 3 and 6 were found to be the only two endothermic steps. The calculation results show that step 6 on the three models have similar reaction energies of about 0.36 eV, which demonstrates that H_2_O desorption in step 6 has a negligible difference among the three N-doped model graphene in the perspective of thermodynamics. Thus, step 3 is considered as the key endothermic step in the catalytic process, which is responsible for the catalytic performances of N-CMNs. As shown in Figure 4(c), the reaction energies of step 3 on the graphitic N model and pyrrolic N model are 0.56 eV and 0.25 eV, respectively, higher than those of C1 (0.21 eV) and C2 (0.20 eV) on the pyridinic N model, indicating that the doping of pyridinic N significantly reduces the endothermic reaction energy of step 3 and boosts the intrinsic POD-like activities of N-CMNs. To further explain the influence of different N-doped species on the reaction activity of step 3, the electronic structural properties of three models and their reaction intermediates were investigated. As shown in Table S5, the Bader charges of structure 1 and H_2_O-adsorbed oxidation-state intermediate 3 were determined. In comparison with other N species, the active site C atom adjacent to pyridinic N has a more positive charge. Besides, the O atom connected to the active site C atom in the intermediate 3 of the pyridinic N model shows less negative charge than that of graphitic and pyrrolic N models, which gives rise to weaker adsorption of H_2_O in intermediate 3 and thus easier desorption of H_2_O during the key endothermic step 3. In addition, as displayed in Figure 4(d), the corresponding difference charge densities of three models further confirm the charge transfer between adsorbed H_2_O and active site in intermediate 3, which also suggests the less negative charge of O atom in pyridinic N model and the weaker interaction between positively charged H atoms in adsorbed H_2_O and O atom. Taken together, pyridinic N dopant is conducive for the generated H_2_O to desorb during the key endothermic step, eventually of great benefit to boost the POD-like activities of N-CMNs.

**Figure 5:**
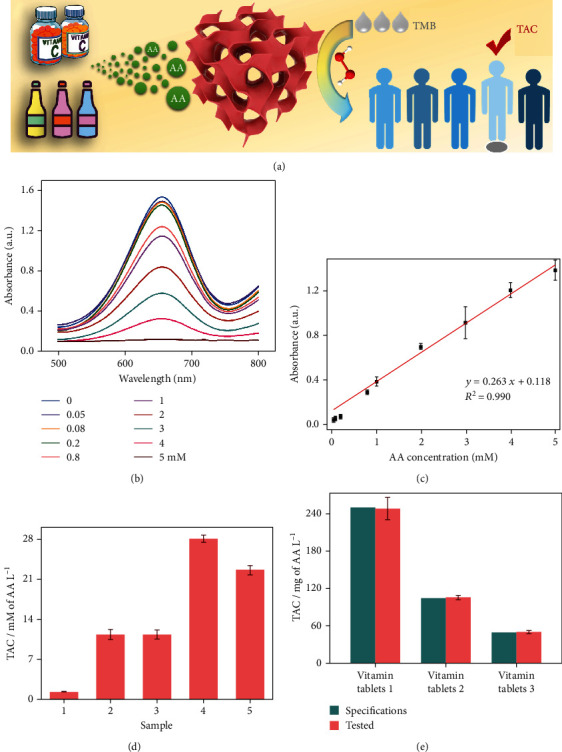
(a) Schematic illustration of the TAC assay using N_P_-CMNs. UV-vis spectra (b) and corresponding calibration curves (c) of the TAC assay with the AA concentrations from 0.05 to 5 mM. TAC of commercial beverages (d) and vitamin tablets (f) (in comparison with the specifications).

TAC is a vital index to assess the quality of medicine and antioxidant food for healthcare [32], which can be identified by detecting the millimolar equivalent of ascorbic acid (AA) L^−1^. Taking advantage of the superior POD-like activity, N_P_-CMNs are beneficial for implementing TAC assay as a concept application (Figure 5(a)). AA, also known as vitamin C, is a common water-soluble micronutrient applied in the prevention and treatment of disease and health care [44, 45]. Given that AA is a strong reducing agent, it can efficiently reduce oxTMB to TMB and consume H_2_O_2_, leading to a decrease in the absorbance of oxTMB [46]. Based on this suggested mechanism, a series of AA concentrations could be monitored (Figure 5(b)), which demonstrated the feasibility of the TAC assay. As shown in Figure S6, the optimized conditions of pH 3.0 and nanozyme amount of 10 *μ*L were adopted for the detection of AA. Accordingly, as displayed in Figure 5(c), a clear linear relationship (*R*^2^ = 0.99) was determined between the absorbance at 652 nm and the concentration of AA ranging from 0.05 to 5 mM, with a limit of detection (LOD) of 0.03 mM (S/N = 3). To further develop the practical use of the assay, the TAC of five commercial beverages (Figure 5(d)) and three vitamin tablets (Figure 5(e)) were detected. The results closely coincide with the specifications from the samples, indicating that the samples meet the quality requirements. Moreover, the potential interferents in food and medicine did not affect the detection (Figure S7), which revealed the robustness of the assay.

## 3. Conclusion

In summary, utilizing the secondary nitrogen doping, we synthesized porous N_P_-CMNs with high pyridinic N content and superior POD-like activity. There is an unexpected finding that the intrinsic POD-like activity of N-CMNs can be modulated by varying the pyridinic N content, revealing that the specific active sites of N-CMNs are relative to the pyridinic N dopant. The mechanism study demonstrated that the pyridinic N dopant renders the active site region less negatively charged in H_2_O-adsorped oxidation-state intermediate in comparison with other N species, which is beneficial to reduce the first H_2_O desorption energy in thermodynamics and boost the intrinsic POD-like activities of N-CMNs. Moreover, the POD-like property of N-CMNs allows them to serve as biocatalysts for bioapplications. Taking advantage of the superior POD-like activity of N_P_-CMNs, a TAC assay was successfully developed, which holds great promise in conducting a quality evaluation of medicine tablets and antioxidant food.

## 4. Materials and Methods

### 4.1. Chemicals and Materials

5, 5-Dimethyl-1-pyrroline-N-oxide, PTA, and TMB were purchased from Aladdin Chemical Reagent Co., Ltd. (Shanghai, China). Glucosamine hydrochloride, dicyandiamide, hydrofluoric acid (HF), acetic acid (HAc), sodium acetate (NaAc), hydrogen peroxide (30%), and AA were obtained from Sinopharm Chemical Reagent Co., Ltd. (Shanghai, China). Colloidal silica 40 wt.% suspension (SiO_2_) was purchased from Sigma-Aldrich. All chemicals were of analytical grade, while solutions were prepared with deionized water (18.2 M*Ω* cm, Millipore).

### 4.2. Instrumentation

The TEM images were carried out on a Titan Themis G2 60-300 (Thermo Fisher, United States). Nitrogen physisorption isotherms were measured on a TriStar II 3flex instrument. XRD patterns were obtained utilizing a D8 ADVANCE (Bruker, Germany). XPS was performed by Thermo ESCALAB 250XI (Thermo Fisher, United States) with an X-ray source (Mg K*α* h*υ* = 1486.6 eV). Raman spectra were measured by a Renishaw inVia Raman spectrometer (Thermo Fisher DXRxi). All the UV-vis absorption spectra were performed by a microplate reader (Tecan Spark, Switzerland). The ESR spectra were obtained from an ESR spectrometer (Bruker EMX plus, Germany).

### 4.3. Synthesis of N-CMNs

Typically, 5 g of glucosamine hydrochloride was dissolved in 20 mL of colloidal silica suspension (2 g SiO_2_). After stirred for 30 min, the derived solution was then freeze-dried. The obtained powder was annealed under flowing N_2_ with a heating rate of 5 °C min^−1^ to 900 °C, and the temperature was kept for 2 h. After that, hierarchically porous N-CMNs-1 were obtained after etching for 12 h by HF (10 wt.%) which can remove SiO_2_ templates, followed by drying at 60 °C for 12 h. N_P_-CMNs were obtained taking dicyandiamide as secondary nitrogen sources. In brief, 500 mg of N-CMNs-1 was mixed with 1 g of dicyandiamide by grounding, and the mixture was heated to 900 °C with a heating rate of 3 °C min^−1^. After keeping at 900 °C for 1 h, N_P_-CMNs were procured directly. For comparison, N-CMNs-2 were synthesized in the absence of dicyandiamide.

### 4.4. Measurement of the POD-Like Activity of N-CMNs

The POD-like activity of N-CMNs was measured taking TMB and H_2_O_2_ as substrates. Typically, the steady-state kinetic assays were implemented in HAc-NaAc buffer (0.1 M, pH 3.0) containing N-CMN solution (5 *μ*L, 1 mg mL^−1^) as catalysts. The kinetic assays of N-CMNs taking TMB as substrate were conducted by adding H_2_O_2_ (100 mM, 100 *μ*L) and TMB with different concentrations (1, 1.5, 2, 3, 4, 5, 7 mM, 100 *μ*L), while the kinetic assays of N-CMNs taking H_2_O_2_ as substrate were carried out by adding TMB (10 mM, 100 *μ*L) and H_2_O_2_ with different concentrations (20, 25, 30, 40, 50, 60, 70 *μ*M, 100 *μ*L). Then, the absorbance of oxTMB at 652 nm was measured at a regular interval. Subsequently, *K*_*M*_ and *V*_max_ were determined by the Michaelis-Menten curve.

### 4.5. DFT Calculations

The catalytic mechanism was studied by DFT calculations which were performed using the Vienna Ab initio Simulation Package (VASP) [47, 48]. The interactions between ion cores and electrons were described employing the Projector Augmented Wave (PAW) [49], and the exchange-correlation energy was computed using the Perdew, Burke, and Ernzerhof functional (PBE) [50]. The plane wave basis sets with a cutoff energy of 400 eV were used. The Brillouin zone sampling was restricted to the *Γ* point with K-point meshes 1 × 1 × 1. The conjugate gradient method was used for geometry optimizations, and the maximum forces on all atoms were smaller than 0.05 eV Å^−1^, while the energy convergence criterion of the electronic step was set to 10^−4^ eV. The structures of all reactant complex, reaction intermediate, and product complex that exist in POD-like catalytic reaction on N-doped model graphene were optimized with lattice constants of *a* = 23.00 Å, *b* = 11.00 Å, and *c* = 26.00 Å. All atoms were allowed to relax. Bader charge analysis was performed to investigate the electronic structural properties [51].

The H_2_O desorption energies (*Δ*E) on intermediate 3 were calculated by:

(7) $$\begin{array}{c} ∆E=E_{\left(\text{oxi}\right)}+E_{\left({\text{H}}_{2}\text{O}\right)}-E_{\left(\text{oxi}+{\text{H}}_{2}\text{O}\right)}, \end{array}$$

where *E*_(oxi)_, *E*_(H_2_O)_, and *E*_(oxi + H_2_O)_ are the energies of intermediate 4, H_2_O molecule in the gas phase, and H_2_O-adsorbed intermediate 3.

The difference charge density of the three models was defined as:

(8) $$\begin{array}{c} ∆\rho =\rho_{\mathrm{int}-3}-\rho_{\text{oxi}}-\rho_{{\text{H}}_{2}\text{O}}, \end{array}$$

where *ρ*_int−3_, *ρ*_oxi_, and *ρ*_H_2_O_ stand for the charge densities of intermediate 3, oxidation-state intermediate without H_2_O adsorption, and adsorbed H_2_O molecule, respectively.

### 4.6. TAC Assay

In a typical procedure, N_P_-CMNs (10 *μ*L, 1 mg mL^−1^), HAc-NaAc buffer (150 *μ*L 0.1 M, pH 3.0), H_2_O_2_ (50 *μ*L, 100 mM), TMB (50 *μ*L, 5 mM), and 50 *μ*L of AA with a series of concentrations were incubated for 5 min, followed by measuring the absorbance of oxTMB (652 nm) in the solution. The same protocol was carried out to determine the TAC of practical samples, including commercial beverages and vitamin tablets, after adjusting their concentration within the detection range. The selectivity of the assay was investigated by interference, including metal ions, carbohydrates, and amino acids. The concentration of AA was 4 mM, while the concentrations of the interferences were 100 mM.

## Supplementary Materials

Supplementary Materials Figure S1. TEM images and HRTEM images (inset) of N-CMNs-1 (a) and N-CMNs-2 (b). Figure S2. XPS spectra of N-CMNs-1, N-CMNs-2 and N_P_-CMNs. Figure S3. The steady-state kinetics of N-CMNs (a-d: N-CMNs-1, e-h: N-CMNs-2, i-l: N_P_-CMNs) toward various concentrations of H_2_O_2_ and TMB, respectively. Figure S4. pH (a) and temperature (b)-dependent activity for N_P_-CMNs and horseradish peroxidases. Figure S5. (a) ESR spectra of free radicals produced in the catalytic processes. (b) Fluorescence spectra of the solution with PTA, PTA+H_2_O_2_, N_P_-CMNs+PTA+H_2_O_2_. Figure S6. Absorbance of reaction systems in the absence and presence of ascorbic acid (AA) varied with pH (a) and nanozymes amount (b). Figure S7. Selective detection of TAC assay. Table S1. Comparison of carbon, oxygen and nitrogen contents [atom %] in the studied N-CMNs in this work with others obtained from XPS. Table S2. Surface concentrations of nitrogen species obtained by fitting the N 1s core-level XPS spectra. Table S3. The Michaelis-Menten constant (K_M_) and maximum reaction rate (V_max_) of the N-CMNs for POD-like catalysis. Table S4. Comparison of the kinetic parameters of N-CMNs and other reported nanozymes. Table S5. The Bader charge of atoms in the intermediate (Int) 1 and 3 of N-doped model graphene.
