# Supplementary material for: Fine-Tuning Pyridinic Nitrogen in Nitrogen-Doped Porous Carbon Nanostructures for Boosted Peroxidase-Like Activity and Sensitive Biosensing
Source: Research (Wash D C). 2020 Nov 6;2020:8202584. doi: 10.34133/2020/8202584 (PMC7877393; doi:10.34133/2020/8202584)
Supplement: Supplementary Materials — Figure S1. TEM images and HRTEM images (inset) of N-CMNs-1 (a) and N-CMNs-2 (b). Figure S2. XPS spectra of N-CMNs-1, N-CMNs-2 and NP-CMNs. Figure S3. The steady-state kinetics of N-CMNs (a-d: N-CMNs-1, e-h: N-CMNs-2, i-l: NP-CMNs) toward various concentrations of H2O2 and TMB, respectively. Figure S4. pH (a) and temperature (b)-dependent activity for NP-CMNs and horseradish peroxidases. Figure S5. (a) ESR spectra of free radicals produced in the catalytic processes. (b) Fluorescence spectra of the solution with PTA, PTA+H2O2, NP-CMNs+PTA+H2O2. Figure S6. Absorbance of reaction systems in the absence and presence of ascorbic acid (AA) varied with pH (a) and nanozymes amount (b). Figure S7. Selective detection of TAC assay. Table S1. Comparison of carbon, oxygen and nitrogen contents [atom %] in the studied N-CMNs in this work with others obtained from XPS. Table S2. Surface concentrations of nitrogen species obtained by fitting the N 1s core-level XPS spectra. Table S3. The Michaelis-Menten constant (KM) and maximum reaction rate (Vmax) of the N-CMNs for POD-like catalysis. Table S4. Comparison of the kinetic parameters of N-CMNs and other reported nanozymes. Table S5. The Bader charge of atoms in the intermediate (Int) 1 and 3 of N-doped model graphene. [file 8202584.f1.docx]

*Supplemental Material*

Fine-Tuning Pyridinic Nitrogen in Nitrogen-Doped Porous Carbon Nanostructures for Boosted Peroxidase-Like Activity and Sensitive Biosensing

Hongye Yan^†,#^, Linzhe Wang^‡,#^, Yifeng Chen^†^, Lei Jiao^†^, Yu Wu^†^, Weiqing Xu^†^, Wenling Gu^†^, Weiyu Song^‡^*, Dan Du^§^, and Chengzhou Zhu^†^*

^†^Key Laboratory of Pesticide and Chemical Biology of Ministry of Education, International Joint Research Center for Intelligent Biosensing Technology and Health, College of Chemistry, Central China Normal University, Wuhan, 430079, P.R. China

^‡^State Key Laboratory of Heavy Oil Processing, China University of Petroleum, Beijing 102249, P.R. China

^§^School of Mechanical and Materials Engineering, Washington State University, Pullman, Washington, 99164 USA

Correspondence should be addressed to Weiyu Song; songwy@cup.edu.cn and Chengzhou Zhu; czzhu@mail.ccnu.edu.cn

^#^These authors contributed equally to this work

Figure S1. TEM images and HRTEM images (inset) of N-CMNs-1 (a) and N-CMNs-2 (b).

Figure S2. XPS spectra of N-CMNs-1, N-CMNs-2 and N_P_-CMNs.

Figure S3. The steady-state kinetics of N-CMNs (a-d: N-CMNs-1, e-h: N-CMNs-2, i-l: N_P_-CMNs) toward various concentrations of H_2_O_2_ and TMB, respectively.

Figure S4. pH (a) and temperature (b)-dependent activity for N_P_-CMNs and horseradish peroxidases.

Figure S5. (a) ESR spectra of free radicals produced in the catalytic processes. (b) Fluorescence spectra of the solution with PTA, PTA+H_2_O_2_, N_P_-CMNs+PTA+H_2_O_2_.

Figure S6. Absorbance of reaction systems in the absence and presence of ascorbic acid (AA) varied with pH (a) and nanozymes amount (b).

Figure S7. Selective detection of TAC assay.

Table S1. Comparison of carbon, oxygen and nitrogen contents [atom%] in the studied N-CMNs in this work with others obtained from XPS.

| Samples | C atom% | N atom% | O atom% | Ref |
| --- | --- | --- | --- | --- |
| N-CMNs-1 | 88.83 | 6.21 | 4.96 | This work |
| N-CMNs-2 | 89.54 | 5.87 | 4.58 | This work |
| N_P_-CMNs | 89.52 | 5.85 | 4.63 | This work |
| NG@H-MMT | 88.60 | 4.20 | 7.20 | [1] |
| LGPCN-600 | 80.00 | 2.64 | 17.35 | [2] |
| N-PCNSs-3 | 92.04 | 3.37 | 4.58 | [3] |

Table S2. Surface concentrations of nitrogen species obtained by fitting the N 1s core-level XPS spectra.

| Samples | Classification | Graphitic N atom% | Pyridinic N atom% | Pyrrolic N atom% | Oxidized N atom% |
| --- | --- | --- | --- | --- | --- |
| N-CMNs-1 | Relative | 52.3 | 13.3 | 12.8 | 21.5 |
| N-CMNs-1 | Absolute | 3.25 | 0.828 | 0.796 | 1.34 |
| N-CMNs-2 | Relative | 50.6 | 14.2 | 14.0 | 21.3 |
| N-CMNs-2 | Absolute | 2.97 | 0.832 | 0.820 | 1.25 |
| N_P_-CMNs | Relative | 50.0 | 17.4 | 11.4 | 21.2 |
| N_P_-CMNs | Absolute | 2.93 | 1.02 | 0.667 | 1.24 |

Table S3. The Michaelis-Menten constant (K_M_) and maximum reaction rate (V_max_) of the N-CMNs for POD-like catalysis.

| Catalyst | [C]  (mg mL^-1^) | Substrate | K_M_  (mM) | V_max_  (10^-7^ M s^-1^) |
| --- | --- | --- | --- | --- |
| N-CMNs-1 | 0.02 | H_2_O_2_ | 17.6 | 3.61 |
| N-CMNs-1 | 0.02 | TMB | 9.52 | 6.29 |
| N-CMNs-2 | 0.02 | H_2_O_2_ | 31.6 | 7.46 |
| N-CMNs-2 | 0.02 | TMB | 19.1 | 6.40 |
| N_P_-CMNs | 0.02 | H_2_O_2_ | 38.7 | 12.6 |
| N_P_-CMNs | 0.02 | TMB | 12.4 | 7.63 |

Table S4. Comparison of the kinetic parameters of N-CMNs and other reported nanozymes.

| Nanozyme | Substrate | K_M_ (M) | V_max_ (10^-7^ M s^-1^) | Reference |
| --- | --- | --- | --- | --- |
| N-CMNs-1 | H_2_O_2_ | 1.76×10^-2^ | 3.61 | This work |
|  | TMB | 9.52×10^-3^ | 6.29 |  |
| N-CMNs-2 | H_2_O_2_ | 3.16×10^-2^ | 7.46 |  |
|  | TMB | 1.91×10^-2^ | 6.40 |  |
| N_P_-CMNs | H_2_O_2_ | 3.87×10^-2^ | 12.6 |  |
|  | TMB | 1.24×10^-2^ | 7.63 |  |
| BN-rGO sheets | H_2_O_2_ | 3.90×10^-2^ | 6.01 | [4] |
|  | TMB | 1.50×10^-4^ | 5.73 |  |
| Pd-Ir cubes | H_2_O_2_ | 3.40×10^-1^ | 0.51 | [5] |
|  | TMB | 1.30×10^-4^ | 0.65 |  |
| PtFe@Fe_3_O_4_ | H_2_O_2_ | 5.36×10^-2^ | 1.08 | [6] |
|  | TMB | 2.13×10^-4^ | 0.55 |  |
| rGO sheets | H_2_O_2_ | 1.54×10^-1^ | 0.71 | [7] |
|  | TMB | 2.32×10^-2^ | 0.87 |  |
| HRP | H_2_O_2_ | 3.7×10^-3^ | 0.87 | [8] |
|  | TMB | 4.3×10^-4^ | 1.00 |  |

Table S5. The Bader charge of atoms in the intermediate (Int) **1** and **3** of N-doped model graphene.

| Model graphene | Atom | Bader charge / e |
| --- | --- | --- |
| Pyridinic N-doped | Int **1** N | 1.907 |
|  | Int **1** C1 | -0.499 |
|  | Int **1** C2 | -0.304 |
|  | Int **3** μ-O | 0.835 |
| Graphitic N-doped | Int **1** N | 1.149 |
|  | Int **1** C1 | -0.317 |
|  | Int **1** C2 | -0.301 |
|  | Int **1** C3 | -0.292 |
|  | Int **3** O | 1.097 |
| Pyrrolic N-doped | Int **1** N | 1.157 |
|  | Int **1** C1 | -0.244 |
|  | Int **1** C2 | -0.290 |
|  | Int **3** μ-O | 0.873 |

Reference

[1] W. Ding, Z. Wei, S. Chen, X. Qi, T. Yang, J. Hu, D. Wang, L.-J. Wan, S. F. Alvi and L. Li, "Space-confinement-induced synthesis of pyridinic- and pyrrolic-nitrogen-doped graphene for the catalysis of oxygen reduction," *Angewandte Chemie International Edition,* vol. 52, no. 45, pp. 11755-11759, 2013.

[2] Q. Niu, K. Gao, Q. Tang, L. Wang, L. Han, H. Fang, Y. Zhang, S. Wang and L. Wang, "Large-size graphene-like porous carbon nanosheets with controllable N-doped surface derived from sugarcane bagasse pith/chitosan for high performance supercapacitors," *Carbon,* vol. 123, pp. 290-298, 2017.

[3] K. Fan, J. Xi, L. Fan, P. Wang, C. Zhu, Y. Tang, X. Xu, M. Liang, B. Jiang, X. Yan and L. Gao, "In vivo guiding nitrogen-doped carbon nanozyme for tumor catalytic therapy," *Nature Communications,* vol. 9, no. 1, p. 1440, 2018.

[4] M. S. Kim, S. Cho, S. H. Joo, J. Lee, S. K. Kwak, M. I. Kim and J. Lee, "N- and B-codoped graphene: a strong candidate to replace natural peroxidase in sensitive and selective bioassays," *ACS Nano,* vol. 13, no. 4, pp. 4312-4321, 2019.

[5] X. Xia, J. Zhang, N. Lu, M. J. Kim, K. Ghale, Y. Xu, E. McKenzie, J. Liu and H. Ye, "Pd–Ir core–shell nanocubes: a type of highly efficient and versatile peroxidase mimic," *ACS Nano,* vol. 9, no. 10, pp. 9994-10004, 2015.

[6] S. Li, L. Shang, B. Xu, S. Wang, K. Gu, Q. Wu, Y. Sun, Q. Zhang, H. Yang, F. Zhang, L. Gu, T. Zhang and H. Liu, "A nanozyme with photo-enhanced dual enzyme-like activities for deep pancreatic cancer therapy," *Angewandte Chemie International Edition,* vol. 58, no. 36, pp. 12624-12631, 2019.

[7] M. S. Kim, J. Lee, H. S. Kim, A. Cho, K. H. Shim, T. N. Le, S. S. A. An, J. W. Han, M. I. Kim and J. Lee, "Heme cofactor-resembling Fe–N single site embedded graphene as nanozymes to selectively detect H_2_O_2_ with high sensitivity," *Advanced Functional Materials,* vol. 30, no. 1, pp. 1905410, 2020.

[8] L. Gao, J. Zhuang, L. Nie, J. Zhang, Y. Zhang, N. Gu, T. Wang, J. Feng, D. Yang, S. Perrett and X. Yan, "Intrinsic peroxidase-like activity of ferromagnetic nanoparticles," *Nature Nanotechnology,* vol. 2, no. 9, pp. 577-583, 2007.
